# 白藜芦醇抑制EGF诱导人肺腺癌A549细胞侵袭

**DOI:** 10.3779/j.issn.1009-3419.2010.04.03

**Published:** 2010-04-20

**Authors:** 宁宇 黄, 宏 芦, 丽君 常, 红伟 张, 灏 张, 冠武 李

**Affiliations:** 1 515041 汕头，汕头大学医学院肿瘤分子生物学开放实验室 Open Laboratory for Tumor Molecular Biology, Shantou University Medical College, Shantou 515041, China; 2 515041 汕头，汕头大学医学院肿瘤研究中心/肿瘤医院中西医结合科 Cancer Research Center/Combination of TCM and Western Medicine of Tumor Hospital, Shantou University Medical College, Shantou 515041, China

**Keywords:** 白藜芦醇, ERK1/2（细胞外信号调节激酶1/2）, PI3K（磷脂酰肌醇三羟基激酶）, Resveratrol, ERK1/2 (extracellular signal regulated kinase 1/2), PI3K (phosphatidyl inositol 3-kinase)

## Abstract

**背景与目的:**

侵袭转移是人肺腺癌死亡的主要原因，表皮生长因子（epidermal growth factor, EGF）可通过活化ERK和PI3K-Akt信号通路，促进人肺腺癌A549细胞侵袭。本实验旨在研究白藜芦醇抑制EGF诱导体外A549细胞侵袭的作用，并初步探讨其机制。

**方法:**

采用MTT法确定白藜芦醇对A549细胞无明显毒性的浓度范围，并以该浓度范围内的白藜芦醇作用EGF诱导的A549细胞，Transwell小室法检测其抑制细胞侵袭作用，明胶酶谱法分析细胞的MMP-2活性变化，Western blot检测EGF信号通路相关蛋白表达。

**结果:**

30 μM内的白藜芦醇无明显的细胞毒性，20 μM处理的受EGF诱导的A549细胞侵袭能力明显降低，MMP-2分泌减少，胞内p-ERK1/2和PI3K（0.5 h-6 h）含量下降。

**结论:**

20 μM白藜芦醇可有效抑制A549细胞侵袭，其机制可能是通过抑制ERK通路和PI3K-Akt通路中相关蛋白激活，最终降低MMP-2的分泌。

## Abstract (en)

**Background and objective:**

Invasion and metastasis are the primary causes of death in patients with pulmonary carcinoma. The epidermal growth factor (EGF) stimulates A549 cells invasion greatly through activating ERK and PI3K-Akt signaling pathway. The aim of this study is to elucidate the inhibitory effect of Resveratrol on EGF-induced invasive ability of A549 cells *in vitro* and explore the molecular mechanism.

**Methods:**

The cytotoxicity of Resveratrol was evaluated by methyl thiazolyltetrazolium (MTT) assay. Then, the A549 cells were treated with EGF and non-cytotoxic concentration of Resveratrol. The cells' invasion were detected by Boyden chamber assay; MMP-2 activity was determined by gelatine zymography assay; the changes of the related proteins were detected by Western blot.

**Results:**

Resveratrol was not toxic to A549 cells at the concentration between 0 to 30 μM. The invasion ability of EGF-induced A549 cells was decreased after treatment with 20 μM resveratrol for 24 h, accompanied by the inhibition of MMP-2 secretion. And the levels of p-ERK1/2, PI3K (within 6 h) were suppressed too.

**Conclusion:**

20 μM Resveratrol inhibits A549 cells' invasion possibly through the suppression of the activation of ERK and PI3K-Akt signaling pathways, subsequently exerting inhibitory effect on MMP-2.

肺腺癌是世界上发病率和死亡率都很高的癌症，这主要是由于其具有极强的转移侵袭能力。而转移侵袭的第一步就是分泌基质金属蛋白酶（matrix metalloproteinases, MMPs），降解细胞外基质^[1, 2]^。因此抑制其分泌MMPs是治疗肿瘤的靶点

白藜芦醇（Resveratrol, RSV）是一种由植物合成的对抗外来病原的多酚类化合物，已在多种植物中被发现。早前研究^[3]^显示白藜芦醇有很好的抗肿瘤生物学活性和药理作用，例如可抑制人肺癌、白血病、食管癌、乳腺癌等细胞的生长。虽已有人报道过白藜芦醇对A549细胞的生长抑制作用，但尚未见有关白藜芦醇抑制肺腺癌侵袭的完整报道。因此，本实验以A549细胞为材料，对白藜芦醇抑制其侵袭的作用及机制进行初步研究。

## 1 材料与方法

### 1.1 主要试剂与仪器

白藜芦醇（Sigma公司，纯度99%）用DMSO（二甲基亚砜）溶解，0.22 μm滤膜过滤除菌，贮存液浓度为500 mmol/L，-20 ℃避光保存。DMEM培养基、噻唑蓝（MTT）均购自Sigma公司，胎牛血清为美国Hyclone公司产品，Transwell和Matrigel购自BD公司，明胶购自上海生工生物工程技术服务有限公司，ERK2兔抗人多克隆抗体购自Cell Signaling，PI3K(p85α)兔抗人多克隆抗体购自Santa Cruz公司，p-ERK1/2兔抗人多克隆抗体购自Bioworld，鼠抗人β-actin抗体购自Sigma公司，其它试剂均为国产分析纯。

### 1.2 细胞培养

A549细胞常规培养于含10%胎牛血清、100 U/mL青霉素、100 U/mL链霉素的DMEM培养基中，置37 ℃、饱和湿度、5%CO_2_的培养箱中培养，2天传代一次，取对数生长期细胞用于实验。

### 1.3 MTT法测定白藜芦醇对A549细胞的毒性作用

将A549细胞浓度调整为1×10^5^个/mL，接种于96孔板，每孔100 μL。实验组加入白藜芦醇（0, 10 μmol/L, 20 μmol/L, 30 μmol/L, 40 μmol/L, 50 μmol/L, 75 μmol/L, 100 μmol/L），每组设6个平行孔，同时设DMSO对照组（加入培养液和0.1%DMSO），置培养箱培养24 h后每孔加入MTT（5 mg/mL）10 μL，继续培养4 h后，然后每孔加入150 μL DMSO，室温避光震荡15 min，酶联免疫检测仪检测492 nm波长OD值，结果取6个复孔的均值。计算不同浓度白藜芦醇对细胞生长的抑制率，从而确定其对A549细胞无明显杀伤的浓度范围（细胞活性抑制率大约小于80%）。

### 1.4 侵袭检测

用培养基将Matrigel稀释为200 μg/mL涂到Transwell的上表面，把A549细胞浓度调整到1×10^6^个/mL，加300 μL到Transwell上室，置培养箱4 h待细胞贴壁后，加药如下：①control，②10 ng/mL EGF，③10 ng/mL EGF+10 μM RSV，④10 ng/mL EGF+20 μM RSV（以上均用无血清DMEM配制），Tranwell下室均加上含1%FBS的DMEM。然后置细胞培养箱培养24 h，取出，去掉培养基，以棉签拭去小室滤膜上表面的细胞，用PBS洗后，将小室置于95%乙醇中固定10 min后再用PBS洗5 min两次，苏木素染色2 min-5 min，水洗后倒置于200倍普通显微镜下随机计数5个视野内的膜下面的细胞数。

### 1.5 明胶酶谱法检测不同浓度的白藜芦醇对EGF诱导A549分泌MMP-2的抑制效果

先对A549细胞如下处理：① control，②10 ng/mL EGF，③10 ng/mL EGF+10 μM RSV，④10 ng/mL EGF+20 μM RSV（以上均用无血清DMEM配制），放入培养箱培养24 h后，吸取培养基，用10 kd孔径的超滤离心管离心（6 000 rpm），每碟培养皿的培养基由3 mL浓缩为150 μL，取25 μL样品与相同体积的2×非还原型样品缓冲液在含有1 mg/mL明胶的10%丙烯酰胺凝胶4 ℃下电泳2.5 h，洗脱液（2.5%Triton X-100）室温下洗脱两次，每次30 min，再用超纯水漂洗4次，每次10 min，之后在孵化液（50 mmol/L Tris, 150 mmol/L NaCl, 10 mmol/L CaCl_2_, pH 7.6）孵化48 h，接着在染色液（0.05%考马斯亮蓝R-250，30%甲醇，10%乙酸）染色2 h，最后用脱色液（20%甲醇，10%乙酸，70%超纯水）脱色，得到结果扫描储存，以Bio-Rad Quantity One分析。

### 1.6 Western blot检测ERK和PI3K-Akt通路相关蛋白的表达变化

收集分别受10 ng EGF、10 ng EGF+20 μM RSV作用不同时间段（0.5 h, 1 h, 2 h, 3 h, 6 h）的细胞和对照组细胞。加入适量含PMSF、钒酸钠的RIPA裂解液，于冰上裂解30 min；4 ℃下10 000 rpm/min离心15 min；取上清，即为细胞全蛋白，用BCA法测定蛋白浓度。10%SDS聚丙烯酰胺凝胶电泳分离蛋白，然后转至NC膜上。10%牛奶封闭非特异抗原，加入一抗[兔抗人p-ERK1/2，ERK2，PI3K(p85α)多克隆抗体，鼠抗人β-actin抗体]4 ℃反应过夜，常温洗膜后加入二抗，室温反应1 h。用Pierce公司的Supersignal West Dura Extended Duration Substrate ECL化学发光系统进行检测，将A液与B液等体积混合，配成发光剂，滴在保鲜膜上，将NC膜倒扣于其上，5 min后用另一保鲜膜将NC膜包裹，X线片压片，曝光20 s-5 min，显影2 min，定影2 min。Western blot结果扫描储存，以BioRad Quantity One分析。

### 1.7 统计学分析

各组实验独立重复3次，实验数据用SPSS 13.0统计软件进行处理，实验组与对照组及诱导组的组间差异比较采用*t*检验分析，*P* < 0.05为差异有统计学意义。

## 2 结果

### 2.1 白藜芦醇对A549细胞活力的影响

MTT实验结果显示：A549细胞在0 μM-30 μM白藜芦醇作用24 h后活力无明显变化，这表明该浓度范围的白藜芦醇对A549细胞无毒性。而在40 μM-100 μM白藜芦醇对A549细胞活力有剂量依赖性的抑制作用（图 1）。

**1:**
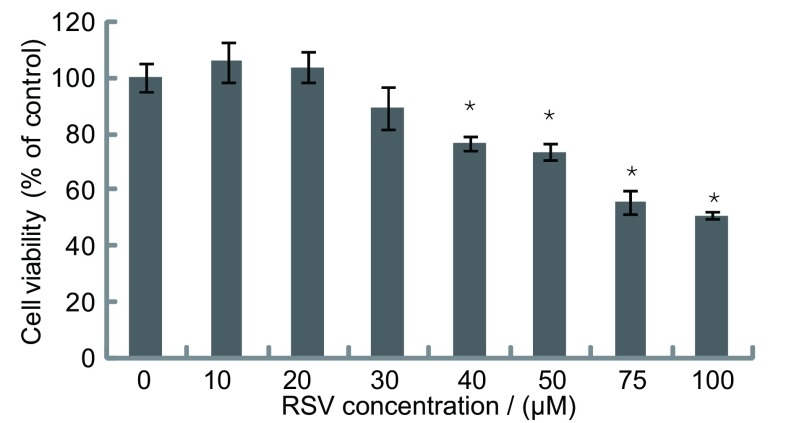
MTT法检测不同浓度白藜芦醇作用24 h对A549细胞活力的影响 A549 cell viability after treatment with Resveratrol at various concentrations for 24 h dectected by MTT

### 2.2 Transwell小室法检测白藜芦醇对EGF诱导A549细胞侵袭的抑制

EGF促进A549细胞侵袭，其侵袭能力增强了22.9%，20 μM白藜芦醇则明显地抑制其诱导细胞侵袭，与E10组相比，其抑制率达到36.4%。而10 μM白藜芦醇对EGF诱导A549细胞侵袭的抑制作用并不明显（图 2）。

**2:**
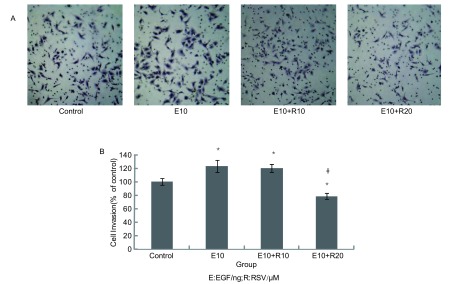
10 μM和20 μM白藜芦醇对EGF诱导A549细胞侵袭的抑制作用 Effects of 10 μM and 20 μM Resveratrol on EGF-induced A549 cells invasion

### 2.3 明胶酶谱法检测白藜芦醇抑制EGF诱导A549细胞分泌MMP-2

EGF促进A549细胞分泌MMP-2，其分泌量比对照组增加了38.6%，10 μM和20 μM白藜芦醇对EGF诱导A549分泌MMP-2均有抑制作用，其中20 μM白藜芦醇效果明显，与E10组相比，其抑制率达到49.3%（图 3）。

**3:**
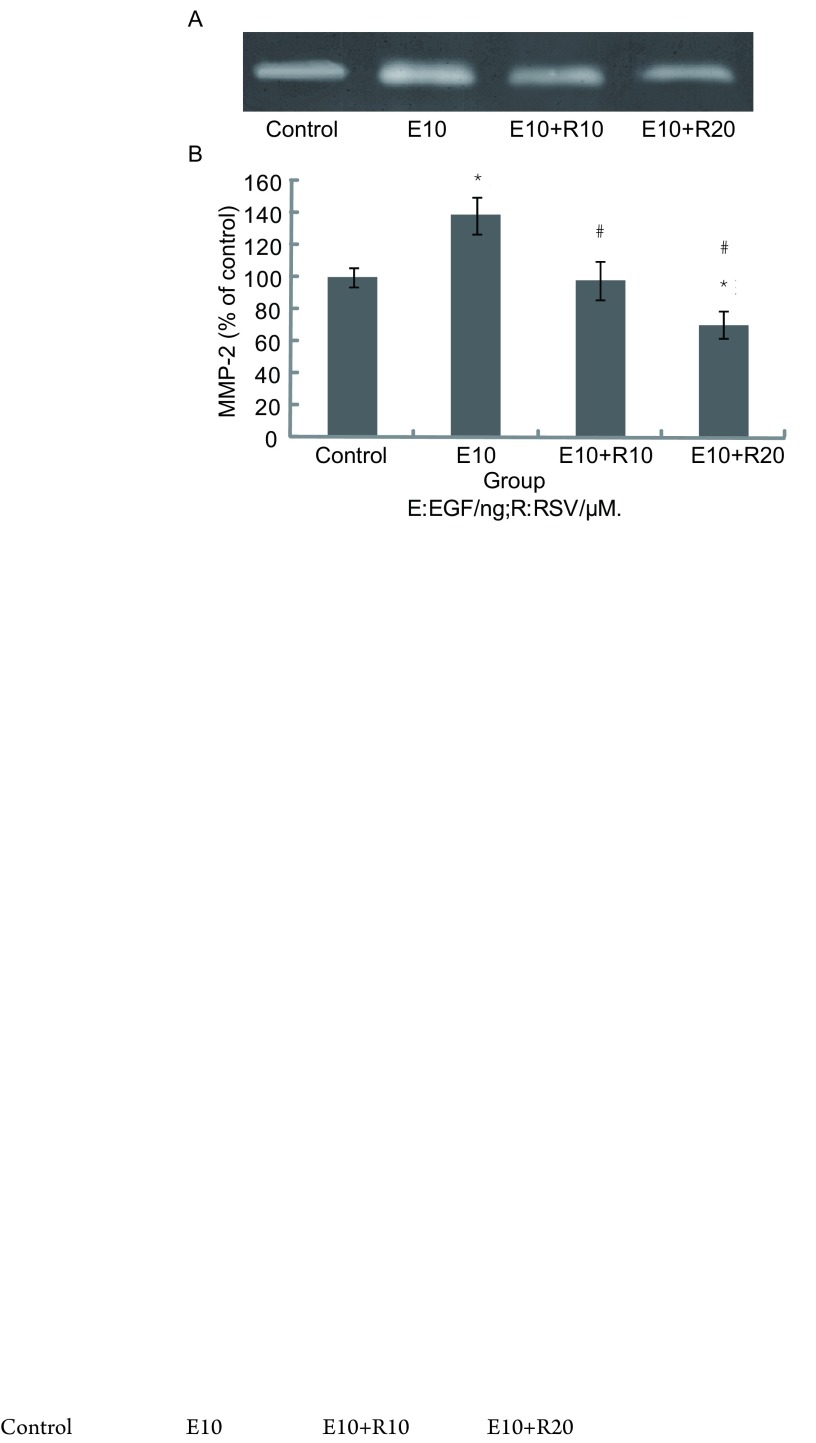
10 μM和20 μM的白藜芦醇对EGF诱导A549分泌MMP-2的抑制作用 Inhibitory effects of 10 μM and 20 μM Resveratrol on the MMP-2 activity of A549 cells stimulated by EGF

### 2.4 20 μM白藜芦醇在不同时间段对p-ERK1/2和PI3K的抑制

EGF均促进了A549细胞中ERK1/2和PI3K的活性，其中对ERK1/2更为显著，特别是在2 h时段，ERR1/2磷酸化程度最高。对比同时段的EGF诱导组，在1 h、2 h、3 h、6 h中，20 μM白藜芦醇在每个时间段中都抑制ERK1/2的磷酸化，类似地，从1 h开始的各个时间段，20 μM白藜芦醇处理组与同时段的EGF诱导组相比，PI3K含量明显下降。（图 4）。

**4:**
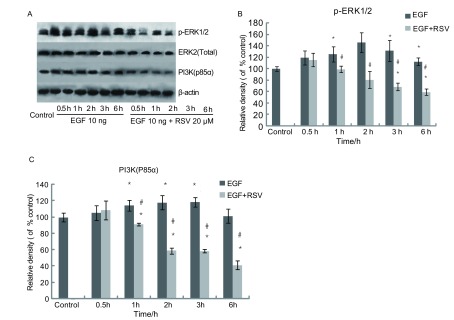
20 μM白藜芦醇对p-ERK1/2和PI3K(p85α)的抑制作用 Inhibitory effects of 20 μM Rresveratrol on p-ERK1/2 and PI3K(p85α)

## 3 讨论

白藜芦醇是一种纯天然小分子化合物，生物作用广泛，近年来其抗癌作用一直受到全世界研究学者的广泛关注。研究集中在白藜芦醇对肿瘤的起始、增殖、发生、转移各个阶段的抑制和诱导肿瘤细胞凋亡，并已发现其对多种肿瘤作用明显。另一方面，白藜芦醇毒副作用小，是一种比较理想抗肿瘤物质。我们通过MTT检测发现白藜芦醇在30 μM之内对A549细胞没有明显的细胞毒性，据此确定用该浓度范围的白藜芦醇对A549细胞的侵袭抑制进行检测。

EGF是一种重要的细胞因子，对细胞的增殖、黏附、转移等起着促进作用。经过多次试验摸索，我们确定10 ng/mL的EGF能明显增强A549细胞侵袭和转移，因此以其作为实验的诱导剂。

恶性肿瘤侵袭周围组织分为三个独立步骤：对细胞外基质（extracellular matrix, ECM）的降解、细胞侵袭转移、细胞增殖^[4]^。MMPs的过度分泌在侵袭的过程中起着关键的作用，肿瘤细胞通过其对细胞外基质进行降解，突破周围组织的限制。之前的文献大多只关注药物对A549过度分泌MMP-2抑制，而不是MMP-9。此外，也有文献^[5]^报道MMP-2和MMP-9在非小细胞肺癌中均会对基底膜有破坏作用，我们经过多次实验发现A549细胞主要分泌MMP-2，而MMP-9相对很少，同时白藜芦醇也只对MMP-2起抑制作用，而对MMP-9没有明显的抑制（未发表资料），因此我们在本研究中只关注MMP-2的变化。那么白藜芦醇为什么只抑制MMP-2，而不抑制MMP-9？有关的研究正在进行，如发现有意义的结果将另文发表。计算EGF诱导组和白藜芦醇处理组中A549细胞侵袭穿透涂有Matrigel小滤膜的细胞数目（Transwell小室法），可以得出，EGF促进了细胞侵袭，而白藜芦醇抑制了这种诱导的侵袭，20 μM白藜芦醇抑制侵袭的效果明显，比EGF诱导组减少36.4%。明胶酶谱法检测发现20 μM白藜芦醇可有效地减少EGF诱导A549分泌MMP-2，比诱导组MMP-2的分泌量减少了49.3%，从而降低了分解、穿透Matrigel的能力，这进一步证实了之前Transwell侵袭实验的结果。

EGF对肿瘤细胞侵袭的促进作用一般认为主要通过ERK通路或PI3K-Akt通路^[6-10]^。这两条信号通路与肿瘤细胞生长、运动、粘附和基质金属蛋白酶分泌与降解等转移因素密切相关。其机制可能是：EGF与表皮生长因子受体（epidermal growth factor receptor, EGFR）结合，EGFR发生二聚化和自身磷酸化，为带有SH2或PTB结构域的胞内信号蛋白分子如Shc、Grb-2、Gab1等提供结合位点。Shc和Grb2聚集到酪氨酸磷酸化的受体上后，鸟苷酸交换因子Sos结合Grb2，活化Ras蛋白，接着通过Ras-Raf-MEK途径将ERK1/2磷酸化为p-ERK1/2，活化的p-ERK1/2后进入核内作用转录因子c-Fos和c-Jun，上调*MMP*基因的表达^[6, 11-14]^。同时，结合到EGFR的Gab1也被活化，其C-端特定酪氨酸磷酸化后成为PI3K的调节亚基p85的结合位点，招募大量的PI3K，在EGF的刺激下PI3K催化PIP2磷酸化形成第二信使PIP3，PIP3促使PDK1磷酸化Akt蛋白的Ser308导致Akt的活化。活化的Akt能提高肿瘤细胞的运动能力、降低细胞间的粘附力、增加核转录因子NF-κB通的活性和MMP的分泌，从而促进细胞侵袭^[6, 10, 15]^。

因此，对ERK通路和PI3K-Akt通路的抑制或者阻断是降低A549细胞侵袭的重要途径。本研究初步探究白藜芦醇抑制EGF诱导的A549细胞侵袭的机制，即用Western blot检测白藜芦醇对A549细胞中ERK1/2和PI3K活化的抑制效果。

Western blot实验结果表明：EGF诱导A549细胞内的ERK1/2磷酸化在短时间内达到很高水平（0.5 h-2 h），之后降低，20 μM白藜芦醇能在各个时间段明显地抑制ERK1/2的磷酸化，尤其是在2 h时间段抑制最为明显，同时检测了ERK2总量，发现基本未发生变化，说明白藜芦醇确实可抑制p-ERK的表达即ERK1/2的磷酸化。同样，白藜芦醇也在大多数时间段中抑制了PI3K，说明白藜芦醇对ERK通路和PI3K-Akt通路均起到了抑制作用。

综合本实验研究结果，我们可以得出：20 μM白藜芦醇能够有效地抑制EGF诱导的A549细胞的侵袭，其机制可能是通过抑制EGFR通路中ERK1/2的磷酸化和PI3K活性，从而降低A549细胞分泌MMP-2，最终抑制其细胞侵袭。
